# Offshore Habitat Preference of Overwintering Juvenile and Adult Black Sea Bass, *Centropristis striata*, and the Relationship to Year-Class Success

**DOI:** 10.1371/journal.pone.0147627

**Published:** 2016-01-29

**Authors:** Alicia S. Miller, Gary R. Shepherd, Paula S. Fratantoni

**Affiliations:** Northeast Fisheries Science Center, National Marine Fisheries Service, 166 Water Street, Woods Hole, MA 02543, United States of America; Department of Agriculture and Water Resources, AUSTRALIA

## Abstract

Black sea bass (*Centropristis striata*) migrations are believed to play a role in overwinter survival and connectivity between juvenile and adult populations. This study investigated oceanographic drivers of winter habitat choice and regional differences between populations of juvenile and adult black sea bass. Trends in cohort strength, as a result of juvenile survival, were also identified. Oceanographic and fisheries survey data were analyzed using generalized additive models. Among the oceanographic variables investigated, salinity was the main driver in habitat selection with an optimal range of 33–35 practical salinity units (PSU) for both juveniles and adults. Preferred temperature ranges varied between juveniles and adults, but held a similar minimum preference of >8°C. Salinity and temperature ranges also differed by regions north and south of Hudson Canyon. Shelf water volume had less of an effect than temperature or salinity, but showed an overall negative relationship with survey catch. The effect of winter conditions on juvenile abundance was also observed across state and federal survey index trends. A lack of correlation observed among surveys in the fall paired with a strong correlation in the spring identifies the winter period as a factor determining year-class strength of new recruits to the population. A rank order analysis of spring indices identified three of the largest year classes occurring during years with reduced shelf water volumes, warmer winter shelf waters, and a 34 PSU isohaline aligned farther inshore. While greater catches of black sea bass in the northwest Atlantic Ocean remain south of Hudson Canyon, the species’ range has expanded north in recent years.

## Introduction

Despite the presence of black sea bass (*Centropristis striata*) in both recreational and commercially important fisheries of the mid-Atlantic Bight (MAB) and southern New England (SNE) [1], knowledge of their ecology remains limited. Black sea bass are a demersal species and laboratory studies have categorized them as euryhaline [2]. Although black sea bass exist as far south as Florida and into the Gulf of Mexico, fish occupying waters north of Cape Hatteras, North Carolina (NC) form a unique stock [3, 4] which exhibits migratory behavior, occuring along the outer continental shelf during the winter, then returning to coastal waters of the inner continental shelf to spawn in late spring. Spawn timing (start and peak) progresses northward, but is more protracted in the southern MAB April–October compared to SNE May–June [5, 6]. Offshore migration in the fall appears to be triggered by bottom water temperatures, between 10–12°C [7, 8], therefore initiating offshore movements earlier in northern latitudes with a progression southward. Fish at the northern extent of the range migrate in a southerly direction as well as offshore, requiring them to travel a greater distance than those to the south in order to reach overwintering areas along the edge of the continental shelf [8]. Although some uncertainty exists on the proximate cause initiating winter migration, feeding ecology (as a result of falling temperatures [9]) and changes in salinity may be potential factors [8]. Recent work by Bell et al. [10] suggests a link between black sea bass distribution on the shelf and bottom temperature. Although tagging data has shown a relationship with temperature, the scope of these studies is limited in time and space [8, 11].

The MAB is the area of the continental shelf extending from Cape Cod, Massachusetts (MA) to Cape Hatteras, NC. The cold, fresh waters that occupy the shelf in this region can be traced poleward to the coast of Labrador [12, 13]. They are delivered to the MAB via a coastal boundary current system that follows the Labrador and Scotian shelves equatorward before entering the Gulf of Maine. Along this path, the composition of the shelf water mass is modified by seasonal heating/cooling, precipitation, river runoff, and mixing with surrounding water masses [14]. In the MAB, the shelf water (SHW) is delineated by a sharp thermohaline front separating the cold, fresh water mass from warmer, more saline slope waters immediately offshore [15]. This so-called shelf-slope front is present throughout the year, wherein temperature and salinity increase dramatically (>5°C and 1–2 practical salinity units (PSU)) over a cross-shelf distance of just 10–40 km [16]. Inshore of the front, the SHW temperature varies considerably in response to annual cycles of heating and cooling; the waters are homogeneous and cold during winter and vertically stratified, with warm water overlying cold, in summer [17]. While there is also a seasonal cycle to freshwater input in the MAB, the distribution of salinity across the shelf remains the same throughout the year; fresh water is consistently observed inshore of more saline water and the 34 PSU isohaline is typically aligned with the center of the shelf-slope front, making it a convenient index for the position of the front and offshore boundary of SHW in the MAB.

From an oceanographic perspective, much attention has been given to the distribution, seasonal evolution, and interannual variability of SHW in the MAB. However, despite being an essential fish habitat for many species of the MAB, there has been little research relative to fisheries ecology in this region. Black sea bass typically congregate along the seaward edge of the SHW during the winter, following their fall offshore migration. The location of this edge proximate to the coast defines the distance necessary for black sea bass to travel to reach their winter residency and can be measured by estimating the volume of the SHW, defined by the 34 PSU isohaline [18, 19]. This boundary undergoes significant seasonal and regional shifts, typically resulting in maximum volumes during the spring in southern New England and late summer in the southern MAB [14].

Large-scale studies of climate change and its effect on distribution of marine fishes has increased the interest for studies focusing on links between environmental processes and their effect on population dynamics and management of fisheries [20, 21]. These processes alter habitat elements and affect species that undergo seasonal migrations, and have been shown to influence juvenile life stages and year-class success [22–25]. In a comprehensive review of winter mortality in fishes, Hurst [26] identifies Hunt [27] as providing the first evidence of overwintering conditions affecting survival of juvenile fishes. Severity of winter temperatures has been correlated with recruitment success of other mid-Atlantic species with inshore spawning behavior [28, 29], and at a population level, has the potential to impact population dynamics by affecting cohort strength. Although temperature is a commonly identified driver [29, 30], other combined factors such as fish size and age (smaller fish undergo greater metabolic stress and more rapid depletion of energy reserves) may also influence overwinter survival in marine fishes [26]. Further, size-dependent mortality has a greater effect on some species at the ends of their geographic range, particularly at northern extremes [31]. Temperate fish populations, like black sea bass, may be more suceptible since they tend to experience the most extreme seasonal temperature variations, particularly in mid-latitudes like the MAB [32].

Hales and Able [22] found 100% mortality in young-of-year (YOY) black sea bass when water temperatures fell to 2–3°C and a likely correlation between seasonal differences in size structure and size-selective winter mortality. Significant variations in oceanographic conditions can occur at the shelfbreak on timescales of days to months caused by a variety of mechanisms, including unstable meanders in the shelf-slope front, slope water intrusions, and/or interactions with warm core Gulf Stream rings [33–35]. For example, extreme temperature anomalies lasting several months have been observed over the continental shelf and attributed to anomalous meanders in the Gulf Stream path [36], while on longer time scales it has been postulated that decadal fluctuations in the position of the shelf-slope front are related to the propagation of salinity anomalies around the North Atlantic [37].

The purpose of this study was to better understand the habitat preference and spatial trends of MAB black sea bass during the overwinter period, identifying any differences that may exist between juveniles and adults and fish originating from the northern and southern extents of the stock. We also examine possible links between these trends and year-class strength, which would identify the importance of the overwinter period and availability of the preferred habitat in juvenile survival.

## Methods

We evaluated the relationship between abundance of black sea bass and environmental conditions using a general additive model (GAM) with a negative binomial distribution. Survey catch was the response variable with spatial and environmental observations considered as explanatory variables. GAMs are a common tool used to investigate the role of environmental factors on fisheries abundance and spatial distribution datasets [38, 39]. Continuous variables (temperature, salinity, and SHW volume) and a regional factor (dividing catch into northern and southern regions of the MAB) were considered as covariates in the model. Fish sampled in this study are excluded from the provisions of the Animal Welfare Act, the US federal law that applies to Northeast Fisheries Science Center (NEFSC) and no permits or licenses were required for collection. Some samples were sacrificed for sex, age, and maturity information in accordance with routine protocols. All other sampled fish were returned to the sea.

**Fig 1:**
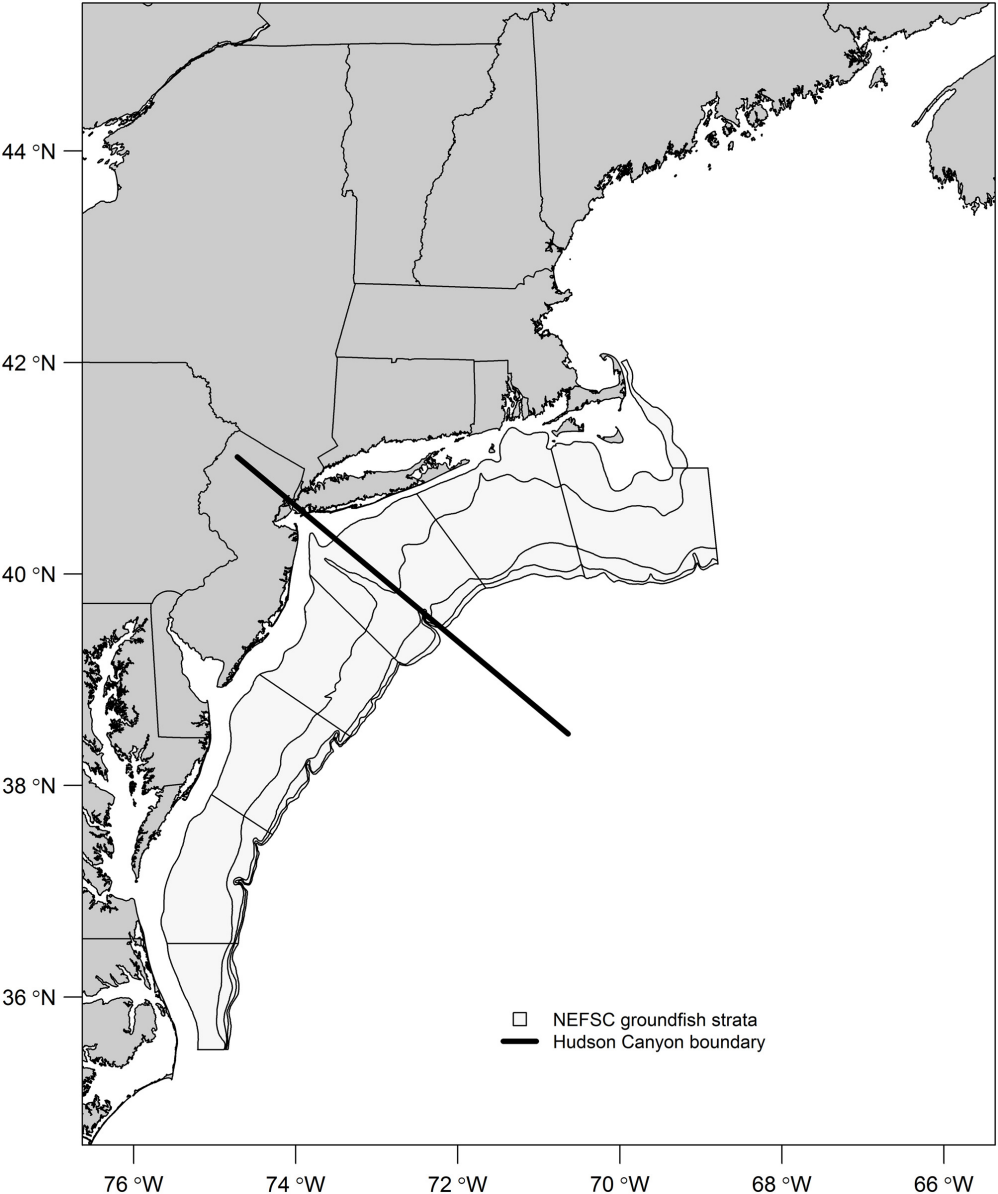
Study area of the NEFSC annual bottom trawl survey with strata used in the black sea bass assessment highlighted. The solid black line marks the Hudson Canyon boundary dividing the region into northern and southern MAB.

Catch data (number per tow) was collected on a tow-by-tow basis from the NEFSC Spring Bottom Trawl Survey with correction factors made after 2009 due to a vessel change [40]. Timing of the spring survey, which commences in late February, is such that it captures a good representation of winter oceanographic conditions and the distribution/abundance of fish during the overwintering period. Temperature and salinity data were collected from CTD casts performed in conjunction with the survey. While bottom temperature data exist since 1992, availability of salinity data limited the GAM analysis to the period 1997–2014. Because black sea bass do not exist throughout the entire spatial extent of the NEFSC survey, only stations from strata included in the stock assessment were used (Fig 1). Shelf water volume was calculated from hydrographic data collected using CTD casts from both NEFSC bottom trawl surveys and ecosystem monitoring surveys from the prior fall (mid September–mid November), the period when black sea bass begin their offshore migration. SHW volume was calculated following [14], where SHW is defined as having a salinity less than 34 PSU.

Separate models were run for juveniles (< = 14 cm, which are age 0 in the fall and age 1 in the spring) and adults (≥14 cm) to achieve a better understanding about how these processes affect different life stages. Models were developed with a stepwise approach using the R statistical software [41] and mgcv package [42]. Cubic regression splines were used for continuous variables with the number of knots (smoothness) set as (k=5) to prevent possible over-parameterization, particularly given that SHW volume is only represented on an annual basis. Regional divisions were explored at both Hudson Canyon and Delaware Bay, two major freshwater outputs that influence oceanographic conditions in the region. A regional factor may be important given that fish at the northern extent exhibit different offshore migration patterns [8] and recent regional differences observed in the fishery north and south of Hudson Canyon that have highlighted potential management issues [43]. The model selection process utilized the Akaike’s information criterion (AIC) statistic when comparing models, assessing the trade-off between more explained deviance and additional parameters (i.e., degrees of freedom).

Annual maps of spring survey catch for juveniles and adults overlaid on temperature and salinity contours were also plotted to explore patterns in spatial distribution with respect to bottom temperature and salinity. Temperature and salinity contours were generated using inverse distance weighting in the gstat package [44], a technique that estimates values based on those at nearby locations weighted by the distance from the interpolation location.

Juvenile abundance was evaluated using relative abundance indices (mean number per tow) from state and federal bottom trawl surveys. A summary of the spatial and temporal extent of these surveys can be found in the most recent stock assessment document [43]. Indices of YOY (age 0) black sea bass were calculated for fall inshore trawl surveys conducted in Massachusetts (MA), Rhode Island (RI), Connecticut (CT), New Jersey (NJ), and Maryland (MD) as well as the fall offshore NEFSC bottom trawl survey, which sampled black sea bass between Cape Cod, MA and Cape Hatteras, NC. Relative abundance indices of age 1 fish (nomenclature assumes a January 1st birthdate) were determined from the same surveys conducted in spring of the following year with the addition of trawl survey indices from Virginia (VA). The time series of indices used in the analysis from MA, RI, and NEFSC was 1984–2012, while CT was 1987–2012, and NJ, MD, and VA were 1989–2012. Rank order of indices within each time series was compared before and after the overwinter period. Spearman’s rank order correlation was conducted across all surveys for both fall and spring.

## Results

**Fig 2:**
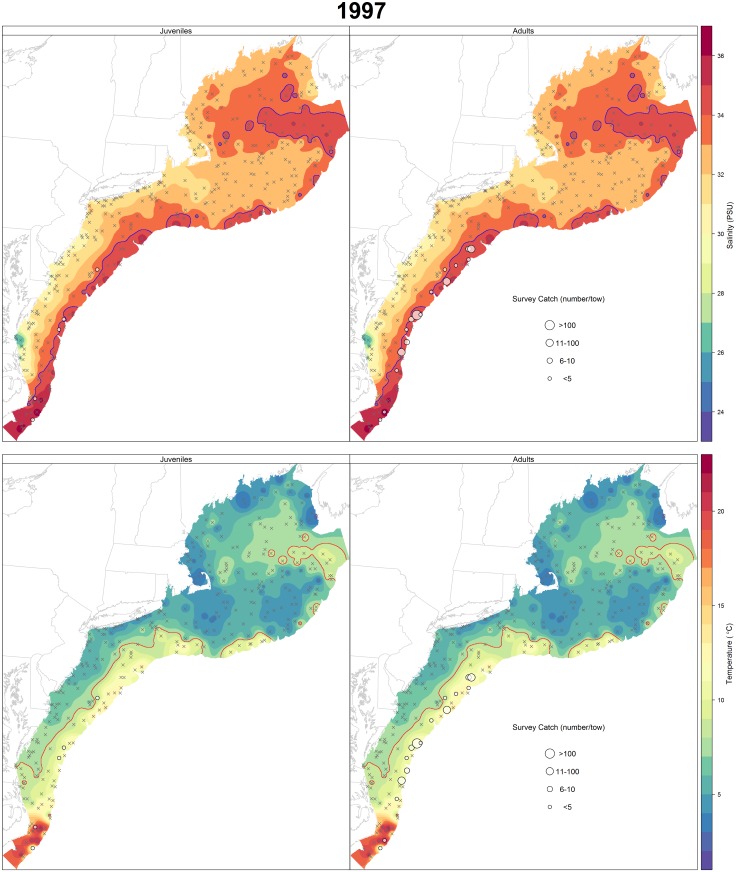
Salinity and temperature contours for 1997 as determined by inverse distance weighted interpolation of the northwest Atlantic Ocean from collected NEFSC survey observations. Survey catch (n) of black sea bass during the NEFSC spring trawl survey of that year is represented by the graduated circles based on the size of the catch. Stations with zero catch are denoted by X. Contour lines highlighting the preferred salinity (34 PSU) and minimum temperature threshold (>8°C) are identified in blue and red, respectively.

**Fig 3:**
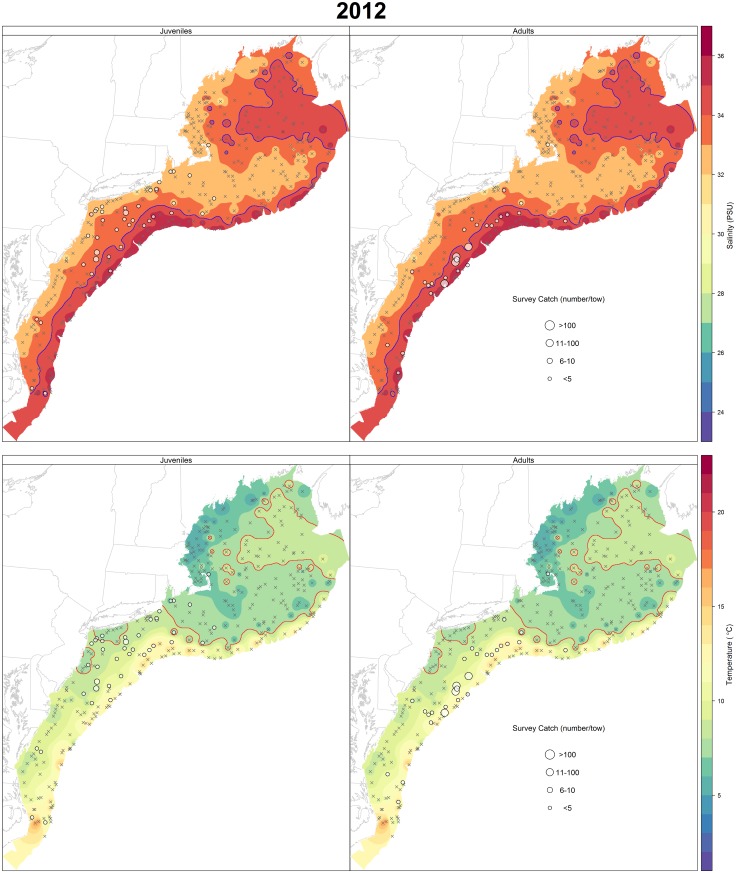
Salinity and temperature contours for 2012 as determined by inverse distance weighted interpolation of the northwest Atlantic Ocean from collected NEFSC survey observations. Survey catch (n) of black sea bass during the NEFSC spring trawl survey of that year is represented by the graduated circles based on the size of the catch. Stations with zero catch are denoted by X. Contour lines highlighting the preferred salinity (34 PSU) and minimum temperature threshold (>8°C) are identified in blue and red, respectively.

A total of 1,817 NEFSC spring survey stations over 18 years were used in the modeling process. Of these stations, there were 187 and 356 with positive catches of juveniles and adults, respectively. Spatial distribution of black sea bass in the early spring confirms the role of temperature and salinity in habitat selection (Figs 2 & 3). Figs 2 and 3 also show that this relationship is consistent across time; the years 1997 and 2012 were chosen as examples that span the length of the time series as well as to highlight an exceptionally warm winter/spring in 2012. Across all years, black sea bass, particularly adults, tend to congregate along the 34 PSU contour, and occupy temperatures greater than 8°C. This affinity is seen to a lesser extent in juveniles which are spread more widely across the shelf. Regional differences north and south of Hudson Canyon are also visible. Higher catches of adults occurred consistently south of Hudson Canyon, although abundance of juveniles and adults has increased at the northern extent of their range in recent years. Prior to 2000, little or no catch of black sea bass occurred north of Hudson Canyon (1968–1999 average catch in the north was 0.09 (n/tow) versus 3.42 (n/tow) in the south). A post-hoc linear regression of year on the northern and southern empirical catch data was performed and revealed a significant positive increase over time for both juveniles (*p* = 0.03) and adults (*p* = 0.003) in the north and no significant difference in the south.

**Fig 4:**
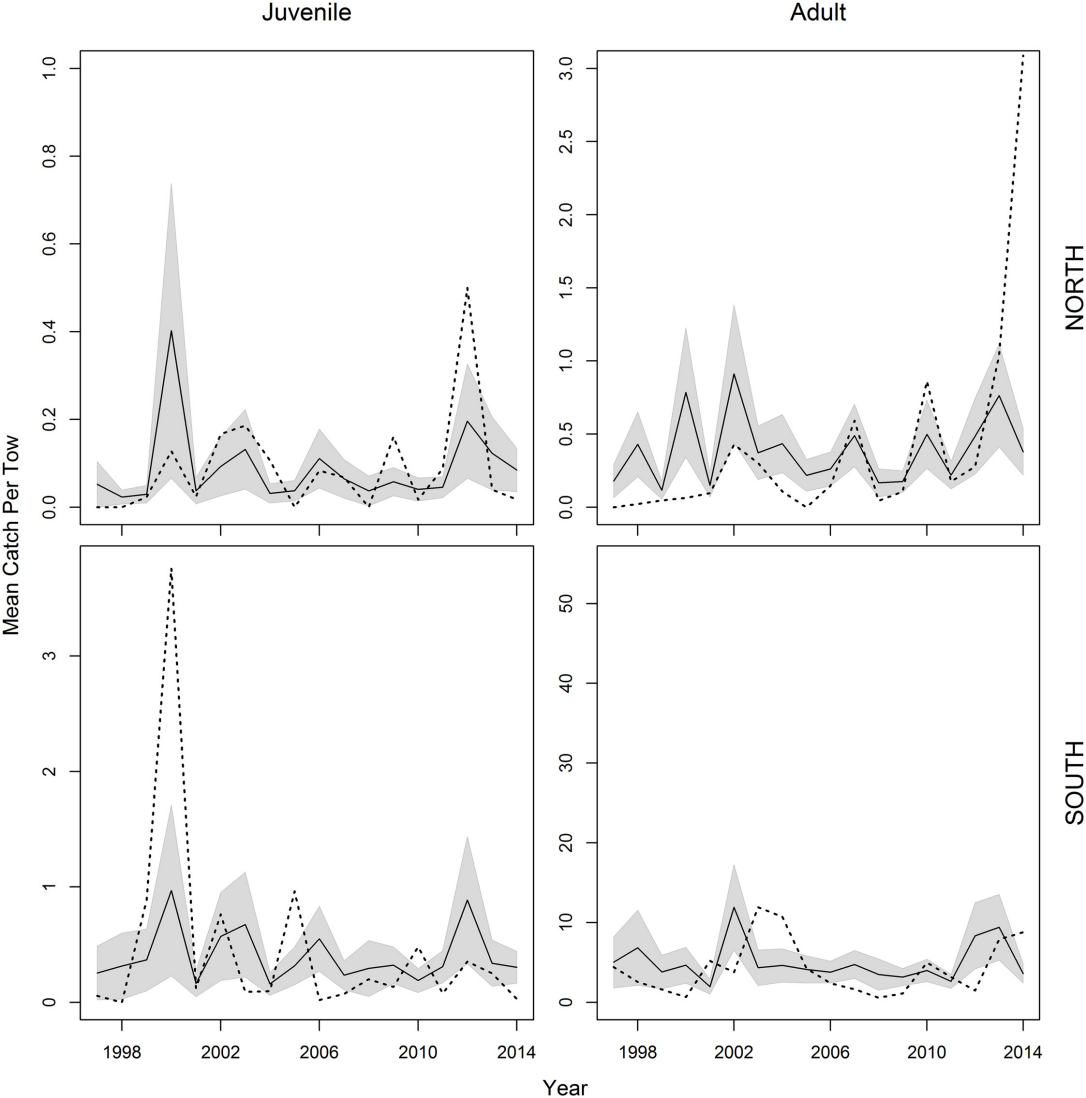
Generalized additive model (GAM) plots identifying estimated effects of smoothed covariates on NEFSC spring survey catch of juvenile black sea bass. The magnitude of the y axis indicates the relative importance of each covariate with the effective degrees of freedom for the smoother denoted in the y axis label. Shaded areas represent 2 SE confidence intervals. Rugplots along the x-axis reflect the relative density of data points.

Generalized additive model results for juvenile black sea bass yielded a final model inclusive of continuous variables temperature, salinity, and SHW volume, with a regional effect on catch that was also included as an interaction term with salinity (Table 1). The final model explained 37.2% of the residual deviance. Results of the individual model runs revealed a better fit when the north-south boundary was placed at Hudson Canyon versus Delaware Bay. The inclusion of a Hudson Canyon split improved the AIC fit and greater catches occurred in the southern MAB. Bottom temperature had a positive linear effect on juvenile catch (Fig 4). Juvenile salinity preference in the north ranged from 32.6–34.7 PSU, peaking at 33.6 PSU. Salinity preference in the southern MAB was shifted slightly higher ranging from 32.9–35.0 PSU, peaking at 34.0 PSU. Shelf water volume had an overall negative relationship with catch. A positive relationship was found between temperature and catch.

**Table 1:** Parameters and estimated factorial and smoother coefficients of the final GAM models for juvenile and adult black sea bass.

**Juveniles**		
**Coefficient**	**Estimate**	**SE**
Intercept	-2.97	0.24
factor(Region) South MAB	0.33	0.35
**Smoother Terms**	**Estimated df**	
*s*(Salinity:North MAB)	2.28	
*s*(Salinity:South MAB)	2.59	
*s*(SHW volume)	3.21	
*s*(Temperature)	0.94	
**Adults**		
**Coefficient**	**Estimate**	**SE**
Intercept	-1.95	0.18
factor(Region) South MAB	0.93	0.28
**Smoother Terms**	**Estimated df**	
*s*(Salinity)	2.77	
*s*(SHW volume)	2.04	
*s*(Temperature):North MAB	0.89	
*s*(Temperature):South MAB	3.81	

**Fig 5:**
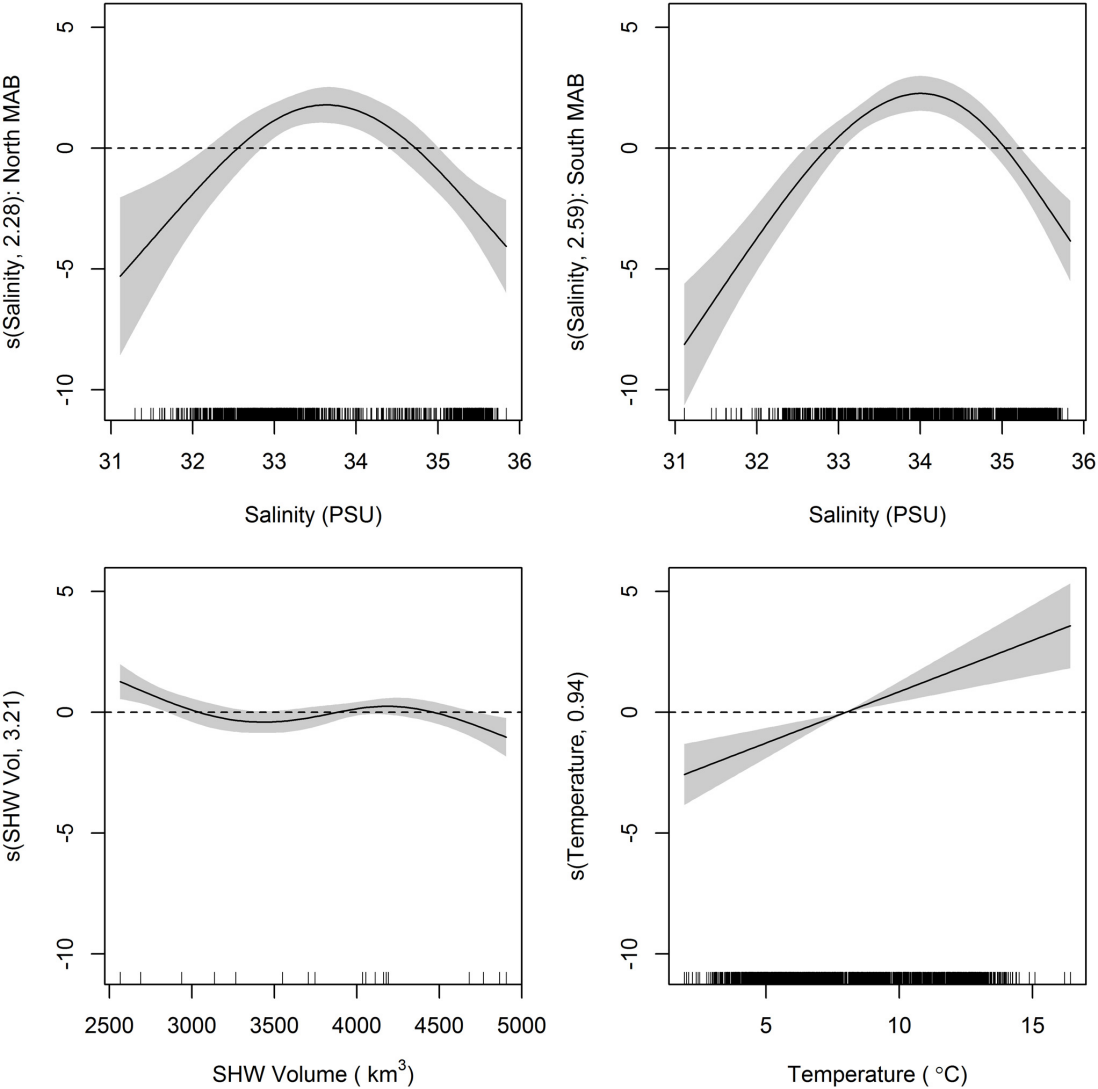
Generalized additive model (GAM) plots identifying estimated effects of smoothed covariates on NEFSC spring survey catch of adult black sea bass. The magnitude of the y axis indicates the relative importance of each covariate with the effective degrees of freedom for the smoother denoted in the y axis label. Shaded areas represent 2 SE confidence intervals. Rugplots along the x-axis reflect the relative density of data points.

Generalized additive model results for adult black sea bass yielded a final model inclusive of continuous variables temperature, salinity, and SHW volume, with a regional effect on catch that was also included as an interaction term with temperature (Table 1). The final model explained 43.0% of the residual deviance. Similar to the juvenile model, Hudson Canyon was the preferred dividing boundary with larger catches in the south, although it had a much greater effect on adults. Shelf water volume had a similar negative relationship with catch, particularly as values increased beyond 4000 km^3^ (Fig 5). Salinity had a similar effect on adults as it did on juvenile black sea bass with preferences that ranged from 32.9–35.0 PSU, peaking at 34.2 PSU. North of Hudson Canyon, temperature had a positive relationship with adult catch with preferred temperatures above 8°C. In the south, temperature preference of adult black sea bass ranged from 7.9°C–15.7°C, peaking at 12.5°C (Fig 5).

The effect of winter survival on juvenile black sea bass abundance was evident in the comparison among indices in the fall compared to spring. Among the fall YOY indices there was low correlation among surveys (Table 2); only the relationship between NEFSC/NJ and RI/CT was significant. The NEFSC survey overlaps the state areas so some correlation would be expected. No clear pattern of strong cohorts emerges from the fall surveys (Table 3). However, among the spring indices of age 1 black sea bass, the relationships change. Adjacent states were significantly correlated, particularly among the states north of Hudson Canyon. As in the fall, the NEFSC survey tends to integrate among the states, which is reflected in the consistent correlation values among surveys north of Virginia (ranging from 0.38–0.53). Among these northern state surveys, the 2011 year-class ranked as first or second highest in the time series (Table 3) and fourth highest for the NEFSC survey. Similarly, the strength of the 1999 and 2001 year-classes were among the top four cohorts in the state time series.

**Table 2:** Spearman rank order correlation coefficients of juvenile black sea bass relative abundance indices from state and federal fall and spring bottom trawl surveys.

**Fall**							
	MD	NJ	CT	RI	MA	NEFSC	
MD	1.00						
NJ	**-0.20**	1.00					
CT	**-0.02**	**-0.13**	1.00				
RI	**0.01**	**0.18**	0.53	1.00			
MA	**-0.09**	**0.02**	**0.04**	**-0.17**	1.00		
NEFSC	**0.21**	0.52	**0.33**	**0.33**	**-0.01**	1.00	
**Spring**							
	VA	MD	NJ	CT	RI	MA	NEFSC
VA	1.00						
MD	0.42	1.00					
NJ	**0.13**	0.42	1.00				
CT	**0.27**	0.54	0.56	1.00			
RI	**0.03**	0.55	0.48	0.86	1.00		
MA	**0.02**	0.40	**0.33**	0.57	0.63	1.00	
NEFSC	**0.23**	0.49	0.53	0.53	0.38	0.41	1.00

Instances where the correlation coefficient was not significant (p>0.05)are in boldface.

**Table 3:** Rank order of relative abundance of juvenile black sea bass from state and federal bottom-trawl surveys.

Fall Age 0							Spring Age 1								
Year/Cohort	MD	NJ	CT	RI	MA	NEFSC	Year	Cohort	VA	MD	NJ	CT	RI	MA	NEFSC
1984			**4**	**4**	22	18	1984	1983					14	15	29
1985			5	5	22	**1**	1985	1984					14	8	19
1986			11	**2**	22	7	1986	1985					7	9	14
1987			18	23	15	19	1987	1986				14	14	15	27
1988		15	14	13	22	28	1988	1987				10	14	15	6
1989		20	27	26	19	25	1989	1988	8	24	21	14	14	15	22
1990	**3**	26	29	21	15	28	1990	1989	**1**	7	25	10	14	14	7
1991	5	11	25	25	5	9	1991	1990	**4**	8	9	8	10	15	21
1992	9	9	23	17	22	10	1992	1991	**3**	13	17	14	14	15	8
1993	23	19	30	30	12	28	1993	1992	21	23	16	14	14	15	28
1994	14	22	15	28	10	24	1994	1993	7	16	18	14	14	15	26
1995	6	16	20	15	22	23	1995	1994	6	**3**	**3**	14	14	11	15
1996	24	6	21	16	15	22	1996	1995	15	25	23	14	14	12	24
1997	11	**1**	28	7	22	**4**	1997	1996	19	11	20	14	10	13	25
1998	18	**2**	24	29	19	11	1998	1997	16	20	24	14	14	15	30
1999	**4**	14	22	20	13	**2**	1999	1998	14	5	14	10	8	15	**3**
2000	8	7	6	**3**	19	12	2000	1999	13	**4**	5	**3**	**2**	**2**	**1**
2001	11	12	10	9	**4**	21	2001	2000	10	9	10	14	14	15	16
2002	**2**	10	8	11	**3**	**3**	2002	2001	**2**	**2**	**1**	**2**	5	**4**	**2**
2003	13	17	7	6	22	17	2003	2002	12	14	12	14	14	15	13
2004	22	**4**	**2**	12	**1**	5	2004	2003	23	21	22	14	14	15	17
2005	20	21	**1**	**1**	9	6	2005	2004	24	22	6	14	14	15	5
2006	10	**3**	19	22	15	13	2006	2005	22	10	19	10	**4**	5	23
2007	7	18	13	8	7	20	2007	2006	17	6	**4**	5	8	15	18
2008	**1**	24	**3**	27	11	14	2008	2007	9	**1**	15	**4**	**3**	**3**	10
2009	18	8	12	24	13	8	2009	2008	18	18	8	6	12	6	11
2010	15	25	9	18	22	16	2010	2009	20	15	13	14	14	7	9
2011	20	13	16	19	8	27	2011	2010	5	19	11	9	13	15	20
2012	15	5	26	10	**2**	15	2012	2011	11	12	**2**	**1**	**1**	**1**	**4**
2013	17	23	17	14	5	26	2013	2012		17	7	7	6	10	12

Top four values within each series are in boldface.

## Discussion

Target measures of biomass estimated during stock assessments include stock-recruitment relationships which are often considered without the influence of external factors; recruitment is not entirely driven by spawning stock biomass alone. This study demonstrates how habitat and oceanographic variables affect population dynamics, potentially influencing the extent of offshore migration and subsequent overwintering success of black sea bass. Incorporating environmental variables into stock assessment processes has become a major area of focus for fisheries scientists and managers. The results of this study provide a perspective on how oceanographic processes on the shelf are affecting black sea bass abundance and spatial distribution.

**Fig 6:**
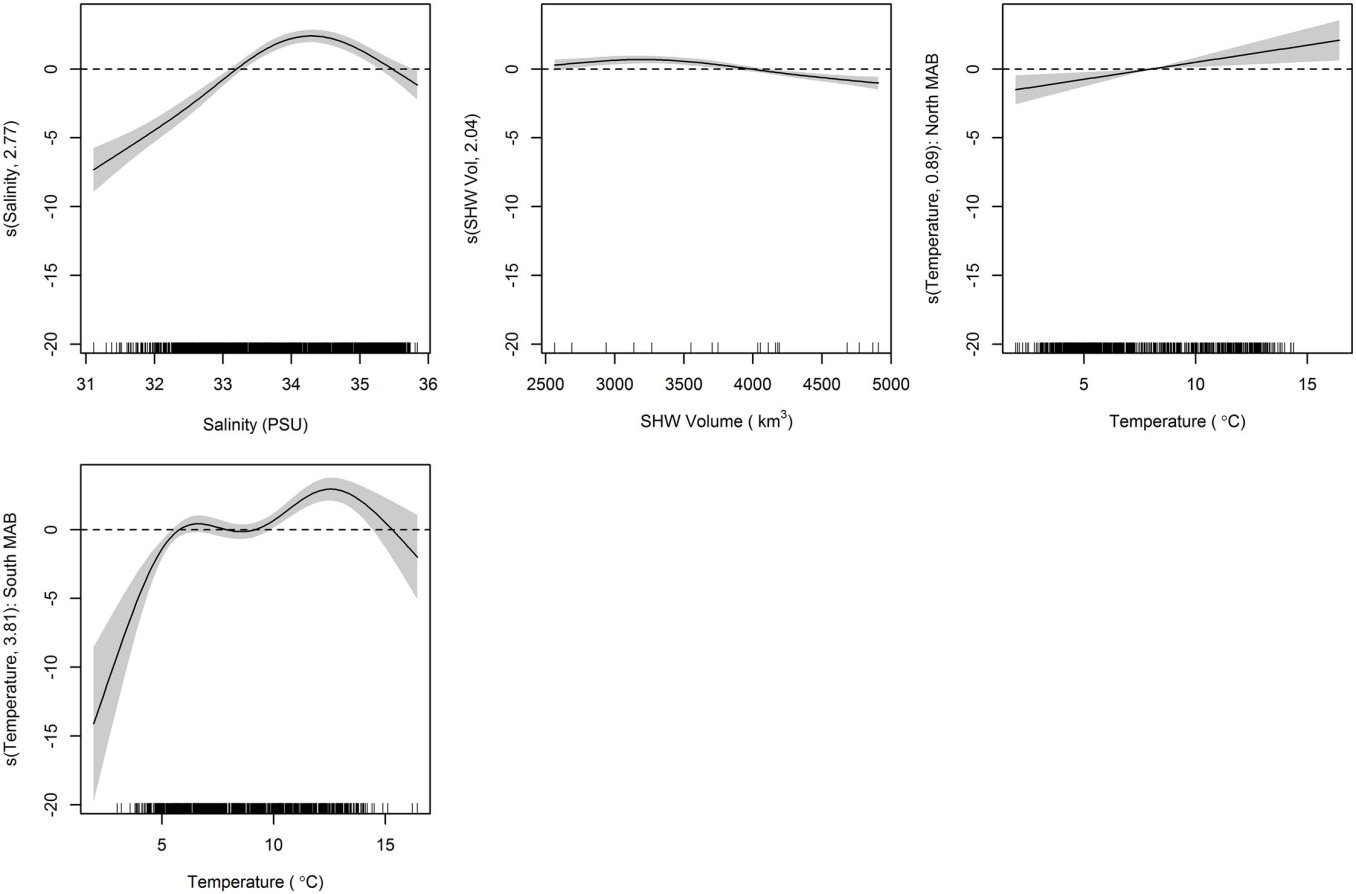
Annual mean catch (n) per tow of black sea bass on the NEFSC spring survey. Data is constrained to survey strata used in the stock assessment. Solid black lines represent model predicted values with 2 standard error confidence intervals shown by the grey surrounding areas. Empirical mean catch of the raw data is denoted by a dotted line.

It is clear that temperature and salinity preference exist in both juvenile and adult black sea bass overwinter habitat choice. Warmer temperatures in the region are sought out by juvenile and adult black sea bass. Because this study focuses on the northern extent of their range, it is likely that maximum temperature thresholds are not reached (i.e. water temperatures never become too warm). This may explain why a linear relationship with no maxima was defined by the model for temperature in the juvenile model and northern MAB of the adult model. Black sea bass showed a more constrained salinity preference, between 33–35 PSU. A slightly higher range resulted for adults and lower range for juveniles in the northern MAB. Given how small these shifts are, they are more likely the result of model fitting than of biological significance. While offshore migration may be triggered by a temperature threshold, salinity seems to be a better driver for overwinter habitat selection. When environmental variables were looked at individually during the model selection process, salinity stood out as a major influence compared to temperature and SHW volume (Table 4). The more simplistic models including salinity as the lone covariate explained 28.4% and 31.4% of the deviance (compared to 17.3% and 19.7% for temperature and 7.4% and 0.9% for SHW volume) for the juvenile and adult models, respectively. Variance described by the final models could be improved upon, particularly for the juvenile model, but estimates fit the data reasonably well (Fig 6).

**Table 4:** Model selection for catch (C) of juvenile and adult black sea bass during the 1997–2014 NEFSC spring survey.

Juveniles				
Model Description	df	Dev (%)	AIC	Δ AIC
null model	1.00	–	1372.1	-126.7
C ∼*s*(Temperature)	4.93	17.3	1320.0	-74.6
C ∼ *s*(Salinity)	3.73	28.4	1272.3	-26.9
C ∼ *s*(SHW volume)	4.19	7.36	1354.5	-109.1
C ∼ RegionDel	2.00	7.0	1351.3	-105.9
C ∼ RegionHud	2.00	8.2	1347.3	-101.9
C ∼ *s*(Salinity) + *s*(Temperature)	5.25	30.6	1265.5	-20.1
C ∼ *s*(Salinity) + RegionHud	4.70	29.2	1270.8	-25.4
C ∼ *s*(Salinity) + *s*(SHW volume)	7.74	32.9	1259.8	-14.3
C ∼ *s*(Salinity) + *s*(SHW volume) + *s*(Temperature)	9.12	35.8	1248.5	-3.1
C ∼ *s*(Salinity) + *s*(SHW volume) + RegionHud	8.75	33.7	1257.8	-12.4
C ∼ *s*(Salinity) + *s*(SHW volume) + *s*(Temperature) + RegionHud	10.02	36.4	1247.2	-1.7
**C** ∼***s*** **(Salinity,RegionHud)** + ***s*** **(SHW volume)** + **s** **(Temperature)** + **RegionHud**	**11.01**	**37.2**	**1245.4**	**–**
C ∼ *s*(Salinity) + *s*(SHW volume,RegionHud) + *s*(Temperature) + RegionHud	8.96	35.1	1251.7	-6.3
C ∼ *s*(Salinity) + *s*(SHW volume) + *s*(Temperature,RegionHud) + RegionHud	11.03	37.1	1245.7	-0.3
C ∼ *s*(Salinity,RegionHud) + *s*(SHW volume,RegionHud) + *s*(Temperature) + RegionHud	11.73	36.4	1250.5	-5.0
C ∼ *s*(Salinity,RegionHud) + *s*(SHW volume) + *s*(Temperature,RegionHud) + RegionHud	13.23	37.1	1249.8	-4.4
**Adults**				
Model Description	df	Dev (%)	AIC	Δ AIC
null model	1.00	–	3640.9	-360.8
C ∼ *s*(Temperature)	4.99	19.7	3497.3	-217.2
C ∼ *s*(Salinity)	3.85	31.4	3388.1	-108.0
C ∼ *s*(SHW volume)	2.80	0.9	3638.3	-358.2
C ∼ RegionDel	2.00	1.4E^−4^	3642.9	-362.7
C ∼ RegionHud	2.00	12.3	3552.1	-272.0
C ∼ *s*(Salinity) + *s*(Temperature)	7.92	35.3	3356.4	-76.3
C ∼ *s*(Salinity) + RegionHud	4.83	37.4	3328.5	-48.4
C ∼ *s*(Salinity) + *s*(SHW volume)	7.51	34.2	3367.0	-86.9
C ∼ *s*(Salinity) + RegionHud + *s*(Temperature)	8.85	39.1	3317.9	-37.8
C ∼ *s*(Salinity) + RegionHud + *s*(SHW volume)	6.99	38.9	3316.0	-35.9
C ∼ *s*(Salinity) + RegionHud + *s*(SHW volume) + *s*(Temperature)	11.16	41.1	3299.6	-19.5
C ∼ *s*(Salinity,RegionHud) + RegionHud + *s*(SHW volume) + *s*(Temperature)	14.01	43.2	3281.3	-1.2
C ∼ *s*(Salinity) + RegionHud + *s*(SHW volume,RegionHud) + *s*(Temperature) + *s*(SHW volume)	14.34	42.5	3289.9	-9.8
**C** ∼***s*** **(Salinity)** + **RegionHud** + ***s*** **(SHW volume)** + ***s*** **(Temperature,RegionHud)**	**12.26**	**43.0**	**3280.1**	**–**
C ∼ *s*(Salinity,RegionHud) + RegionHud + *s*(SHW volume) + *s*(Temperature,RegionHud)	13.98	43.3	3280.4	-0.3
C ∼ *s*(Salinity) + RegionHud + *s*(SHW volume,RegionHud) + *s*(Temperature,RegionHud)	14.15	42.9	3284.8	-4.7

Percent deviance explained (Dev) and Akaike’s information criterion (AIC) are given for each model.

Selected model highlighted in boldface.

It is important to note that the strong salinity constraint demonstrated by the GAM results is not necessarily physiologically driven. It is more likely that the model is highlighting the shelf-slope front as a preferred habitat, since salinity is the leading indicator of frontal position. The front may be preferred by black sea bass for a variety of reasons. For instance, the upwelling circulation within the front makes it a region of high productivity [45–48] and convergent flow in and around the front near the shelfbreak’s abrupt topography may aggregate prey [49–51]. Finally, the thermohaline front is a boundary between two contrasting water masses wherein water properties change rapidly over a very short distance (up to 5°C and 2 PSU over just 10–40 km horizontally). Offshore migrating black sea bass in search of favorable habitat may use the abrupt change in water properties as an indicator of preferred winter habitat.

Since SHW volume is defined based on salinity [14], it is not surprising that both would be related to the distribution of black sea bass. While the effect of SHW volume on catch is less than that of salinity or temperature (likely due in part to the fact that it is estimated at annual and regional scales), its presence in the model does provide some insight into the role of larger oceanographic processes. Both juvenile and adult models show an overall negative trend, particularly as SHW volume levels surpass 4000 km^3^. This suggests that the suitable habitat for migrating black sea bass is larger and extends shoreward during years when shelf water volumes are condensed, resulting in a shorter distance to travel and giving them an energetic advantage to surviving the migration. Years where preferred winter temperature, salinity, and SHW volume (as identified by the GAM models) are observed correspond well with years of good recruitment across spring survey abundance indices. Table 3 highlights the spring years 2000, 2002, 2008, and 2012 as strong age 1 recruitment years. Based on calculations of annual mean temperature and salinity from the NEFSC spring survey, these years were defined as some of the warmest, with higher levels of salinity and lower SHW volume. Some caution should be taken for 2008 since the number of stations was truncated that spring, but 2000 is identified as the lowest SHW volume, 2002 and 2012 are the warmest average temperature, and all three years are among the top five years with highest mean salinity in the time series. While many factors may influence survival of migrating fish, migration distance and fish size and condition have been shown to be of particular importance [52]. In general, fishing pressure has reduced the number of larger, later maturing fish that are capable of longer migrations [52].

Black sea bass had a greater presence in northern waters in the latter years of the study, stretching their range as far as the Gulf of Maine from 2012 onward (Fig 3). Even though black sea bass were not included in the Nye et al. [21] study, shifts in the extents of summer flounder (a species managed in the same complex) distribution were noted. Expansion of the spatial distribution of black sea bass to the north has been recognized among scientists and industry in recent years [10], and this study supports that conclusion. Strong correlation exists among state spring indices (particularly the northern most states), identifying years with strong recruitment of age 1 fish (Table 3). Paired with a lack of correlation of fall indices, this indicates that winter conditions play a decided role in survival of juvenile black sea bass and year-class strength. For example, a strong year-class of YOY black sea bass observed in the fall is not always indicative of a strong year-class of one-year-olds the following spring. Because the northern end of the population experiences a more energetically taxing winter migration, it seems likely that recent warming temperatures in the northwest Atlantic might promote stronger year-classes in SNE for black sea bass. It is common for environmental variables to have the greatest effect on population dynamics at the edge of their geographic range, particularly correlations with recruitment [53]. It is apparent that the occurrences of black sea bass north of Cape Cod, once rare, are becoming more common.

Although the intent of analyzing differences between northern and southern MAB black sea bass was not to focus on identifying discrete stocks, behavioral differences between the regions as a response to environmental conditions were apparent. Results of this study agree with those of Moser and Shepherd [8] and show that migration processes differ for black sea bass originating north and south of Hudson Canyon. This is not surprising given the important differences that exist in the oceanography of these two regions. First, the region south of Hudson Canyon is the recipient of large amounts of freshwater from the Hudson River, Delaware Bay, and Chesapeake Bay estuaries. There are no equivalent proximate freshwater sources in the northern MAB. Second, the coastline in the southern region is oriented parallel to prevailing, summer winds, while the coastline in the northern MAB is oriented more obliquely. The along shelf winds drive significant cross shelf flows in the southern MAB, seasonally advecting freshwater discharge offshore near the surface and driving coastal upwelling in the central MAB [54]. Third, the shelf south of the Hudson Shelf Valley narrows and shoals dramatically. As a result, seasonal heat input at the surface is efficiently redistributed vertically, leading to larger seasonal warming of bottom waters than in the north. This narrow shelf and close proximity of the Gulf Stream provide greater opportunity for exchange and mixing with slope waters offshore. While slope water intrusions are ubiquitous throughout the MAB, their occurrence actually peaks south of Hudson Canyon [35]. Of course, the narrowing of the shelf also means that the shelf-slope front, and hence the overwintering grounds, are closer to shore than they are further to the north.

Model selection and inclusion of covariates was clearer in the adult model (Table 4), partly due to the greater number of positive tows of adults versus juveniles. Positive tows of adult black sea bass outnumbered that of juveniles by over eight times throughout the time series. Juveniles also appear to be more scattered spatially, rather than displaying a strong affinity to any specific temperature or salinity as seen with adult black sea bass (Fig 3). While this was not the case in all years, it does indicate migration differences by age, possibly due to lack of experience or limited swimming speeds of juveniles. Information regarding adult habitat preference is important for understanding requirements of the spawning stock, but further knowledge of recruitment dynamics is needed. A directed survey targeting juvenile black sea bass in the future would be useful.

While the focus of this work was to identify environmental drivers and a preferred oceanographic habitat, these variables make up a small part of what is responsible for the trends seen in black sea bass abundance. Other mechanisms such as food web dynamics, including predator-prey interactions, need to be investigated. Paired with the results of this study, future work could focus on growth and condition of black sea bass in order to further understand overwinter and migration survival, year-class connectivity, and how a changing ocean could affect future populations. Direct sampling of the shelf and slope water boundary of the MAB ecosystem would also illuminate an area that exists as a popular overwintering habitat of many seasonally migratory fishes that remains highly understudied.

This study demonstrates how links can be made between black sea bass abundance, spatial distribution, and oceanographic characteristics on fine and broad scales. Understanding basics such as temperature and salinity preference give us a better perspective of what initially drives black sea bass to migrate and defines their overwintering grounds. Characterizing catches with aspects of the environment is a step towards integrating oceanographic variables into stock assessments and understanding how changes to the ecosystem may affect assessment models. This modeling exercise identified factors that determine habitat preference, spatial trends, and poses further questions regarding regional differences and migratory success of black sea bass. As interest in the ecology the northwest Atlantic shelf mounts, more details of the habitat and its seasonal processes will help answer some of these questions.
