# Correction: Offshore Habitat Preference of Overwintering Juvenile and Adult Black Sea Bass, *Centropristis striata*, and the Relationship to Year-Class Success

**DOI:** 10.1371/journal.pone.0156355

**Published:** 2016-05-19

**Authors:** Alicia S. Miller, Gary R. Shepherd, Paula S. Fratantoni

The images for Figs 4–6 are incorrectly switched. The image that appears as Fig 6 should be Fig 5, the image that appears as Fig 5 should be Fig 4 and the image that appears as Fig 4 should be Fig 6.

**Fig 4:**
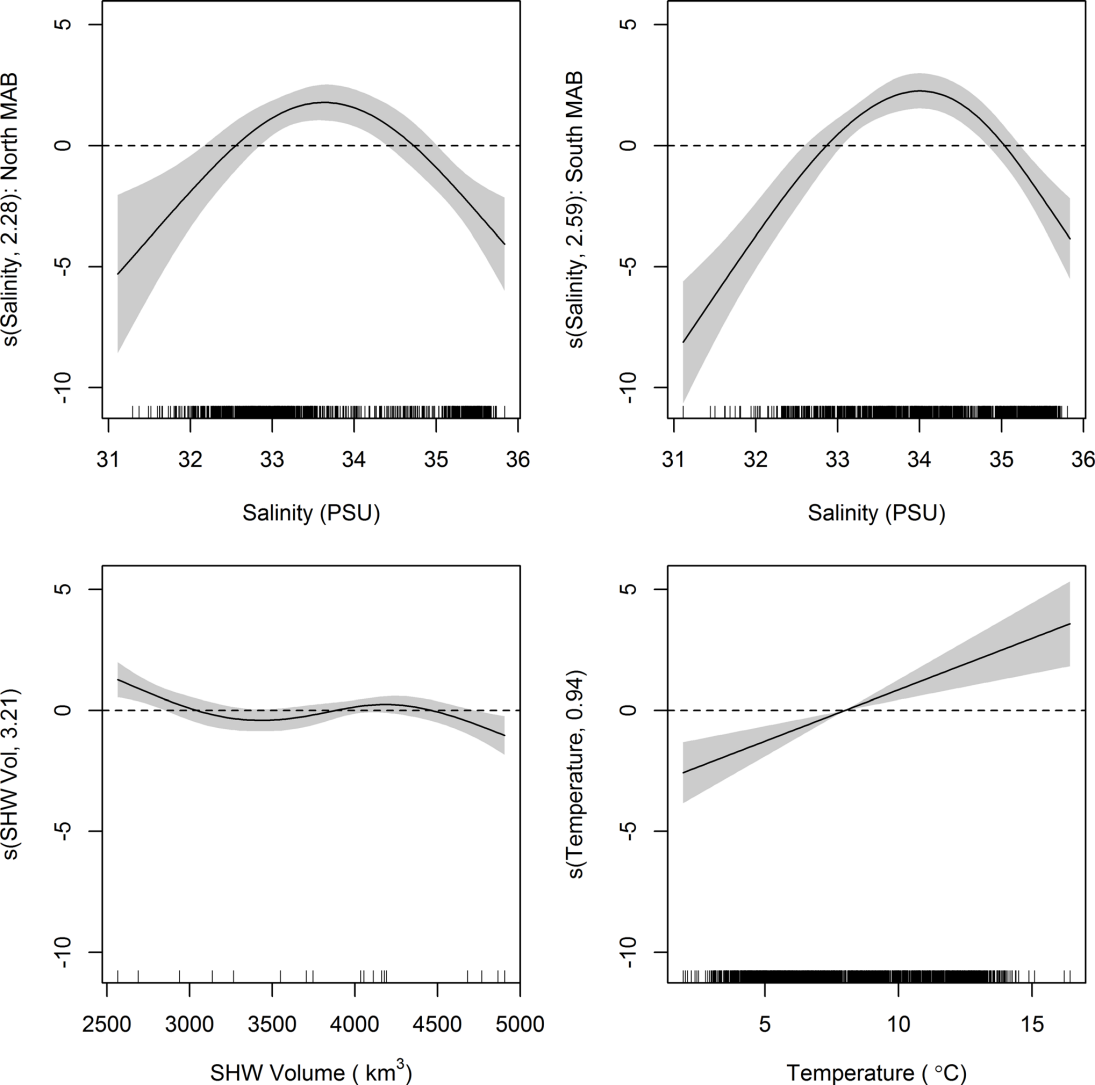
Generalized additive model (GAM) plots identifying estimated effects of smoothed covariates on NEFSC spring survey catch of juvenile black sea bass. The magnitude of the y axis indicates the relative importance of each covariate with the effective degrees of freedom for the smoother denoted in the y axis label. Shaded areas represent 2 SE confidence intervals. Rugplots along the x-axis reflect the relative density of data points.

**Fig 5:**
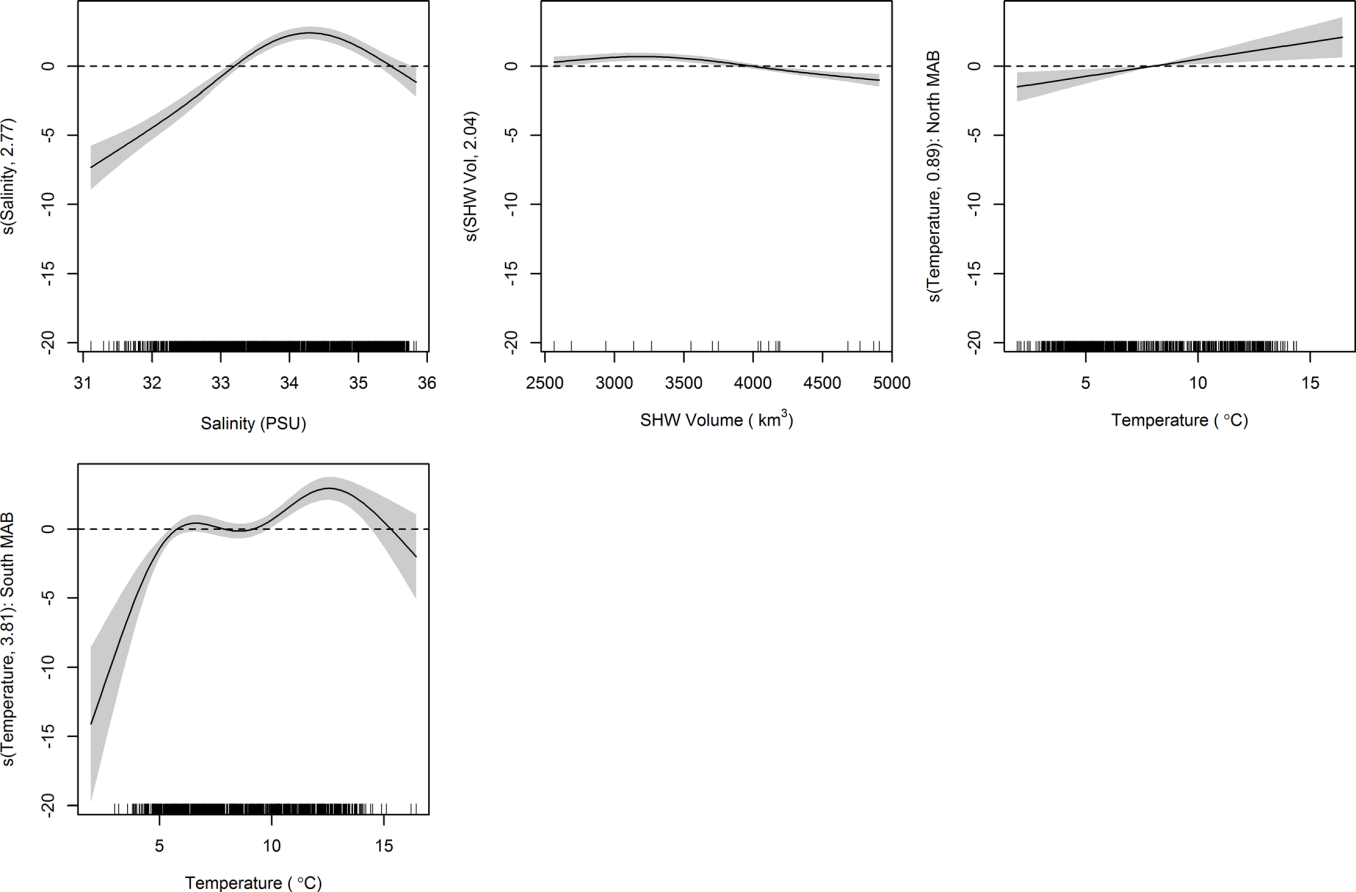
Generalized additive model (GAM) plots identifying estimated effects of smoothed covariates on NEFSC spring survey catch of adult black sea bass. The magnitude of the y axis indicates the relative importance of each covariate with the effective degrees of freedom for the smoother denoted in the y axis label. Shaded areas represent 2 SE confidence intervals. Rugplots along the x-axis reflect the relative density of data points.

**Fig 6:**
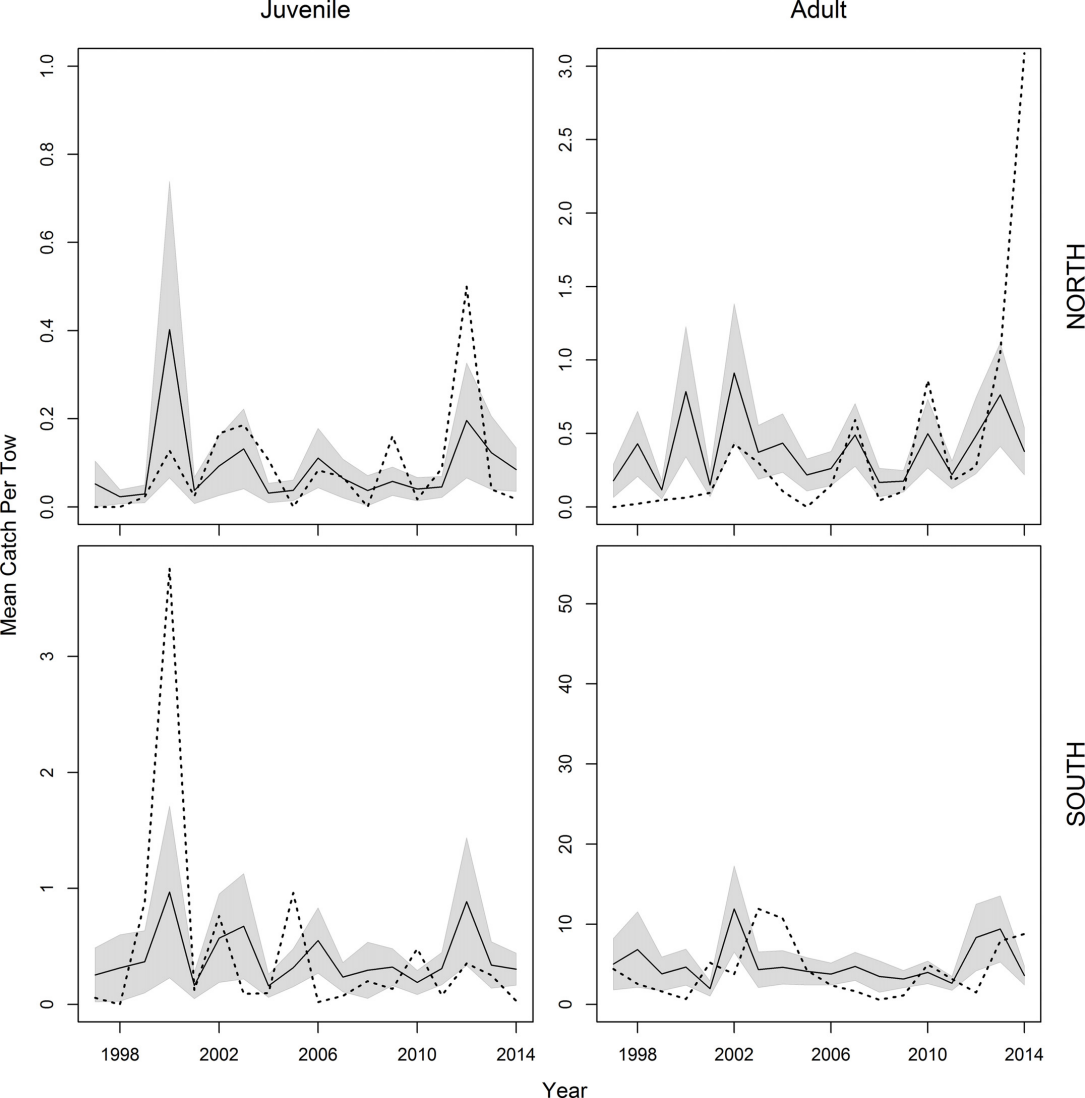
Annual mean catch (n) per tow of black sea bass on the NEFSC spring survey. Data is constrained to survey strata used in the stock assessment. Solid black lines represent model predicted values with 2 standard error confidence intervals shown by the grey surrounding areas. Empirical mean catch of the raw data is denoted by a dotted line.
